# A Qualitative Study to Explore Ways to Observe Results of Engaging Activities in Clients with Dementia

**DOI:** 10.1155/2017/7513875

**Published:** 2017-08-10

**Authors:** Masahiro Ogawa, Seiji Nishida, Haruna Shirai

**Affiliations:** ^1^Department of Human Health Sciences, Graduate School of Medicine, Kyoto University, Kyoto, Japan; ^2^Faculty of Health and Welfare, Prefectural University of Hiroshima, Hiroshima, Japan; ^3^School of Health Sciences, Bukkyo University, Kyoto, Japan

## Abstract

**Background:**

Many occupational therapists face the challenge of helping clients with dementia to select and perform meaningful occupations, which may be difficult due to cognitive impairment. Understanding tacit knowledge of well-experienced occupational therapists could positively affect occupational therapy practice for clients with dementia.

**Objectives of Study:**

To explore the observations of experienced occupational therapists when evaluating the effects of activities in clients with dementia.

**Methods:**

Ten occupational therapists with over 10 years of clinical experience participated in this qualitative study. In-depth interviews were conducted to ask the question, “What do you observe in clients with dementia when you assess the effectiveness of activities among these clients?”* Findings*. From 47 cases, we found five major themes and 18 subthemes. Main themes were “engaging activity,” “emotional expression during activity,” “verbal expression during activity,” “social interaction through activity,” and “something obtained as outcome of activity.”* Relevance to Clinical Practice*. The 18 subthemes could be used as viewpoints to observe engagements of activity in clients with dementia.

**Limitations and Recommendations for Further Research:**

Future studies could examine which viewpoints were utilized for each type of activity and/or severity of dementia as this was not investigated in the current study.

## 1. Introduction

The primary goal of occupational therapy is to engage people in occupation [1]. Successful occupation in an engaging activity by individuals with dementia is associated with positive affective outcomes [2, 3]. For example, observation of nursing home residents with dementia revealed that greater well-being was associated with positive and enjoyable engagement in activities [4]. Perrin [5] indicated that the loss of ability to engage in occupation occurring in progressive dementia is a powerful sign of unmet human needs and a major threat to the health and well-being of clients with dementia. These problems were caused by the difficulty of clients with dementia to choose and engage in occupations. In particular, dementia often deprives individuals of the ability to engage in occupations because of difficulty in occupational choice and linguistic representation about feeling and emotion. This is due to both lack of awareness and cognitive impairments such as memory disturbance, orientation disturbance, loss of decision-making capacity, and lack of language comprehension or expression [6]. Therefore, in occupational therapy, many occupational therapists face the challenge of helping clients to select and perform meaningful occupations.

In clinical settings, well-experienced occupational therapists competently select and facilitate their patients' occupational engagement. They empirically seem to have unconscious viewpoints when observing the subjective experiences, feelings, and emotions expressed by a client while engaging in an activity. Understanding occupational therapists' unconscious judgement could positively affect occupational therapy practice for clients with dementia. Therefore, the question arises: “What are the observations of experienced occupational therapists when evaluating the effects of activities in people with dementia?”

During the past decade, understanding the subjective experience of well-being and quality of life has been recognized to be important when observing people with dementia. [7, 8]. Because clients with dementia have difficulty expressing their feelings and experiences while performing activities, they might also struggle with formal self-report measures that examine the effects of engaging occupations, such as the Canadian Occupational Performance Measure (COPM) [9] and the Aid for Decision-Making in Occupation Choice (ADOC) [10].

Studies analyzing interviews conducted with people with dementia have recommended a combination of observations and interviews [11, 12]. Observational assessment for clients with dementia can be crucial, especially for those with severe dementia. Tomori et al. [13] reported that when using the ADOC, an iPad application allowing the user to choose meaningful activities could not in fact identify meaningful activities for clients with severe dementia because of their inability to express opinions. Therefore, certain observational assessment tools, such as Dementia Care Mapping (DCM) [14, 15], have been developed to improve the well-being of individuals with dementia. Brooker and Duce [16] reported that DCM could be used to assess the impact of therapeutic activity in individuals with dementia. It has been reported that meaningful activities for people with dementia are crucial to their well-being, but it is currently unknown which viewpoints of the therapists' observation enable them to determine clients' well-being. Obtaining clearer and more explicit viewpoints could help us in explaining how clients with dementia engage in activities and to judge whether these activities are meaningful. Therefore, this study examined the observations made by experienced occupational therapists while evaluating the effects of activities in clients with dementia.

As a research methodology, the phenomenological approach focuses on the world of everyday life and pays special attention to the careful description of how the ordinary is experienced and expressed in the consciousness of individuals [17, 18]. Moreover, when collecting data on the experiences of occupational therapists, in-depth interviews can be useful to gain a window into the subjective human experience and to develop insight into how individuals think about the world [19]. Given the characteristics of these methods, the phenomenological approach and in-depth interviews are suitable to examine the observations of occupational therapists when evaluating the effects of activities in clients with dementia.

The objective of this study was therefore to explore the observations of experienced occupational therapists when evaluating effects of activity in clients with dementia using the aforementioned methodology. By clarifying the viewpoints, we aimed to design an observational assessment framework for clients with dementia.

## 2. Methods

### 2.1. Participants

Study participants were experienced occupational therapists who were proficient at verbally expressing their experiences. Participants were recruited via purposive sampling based on the following inclusion criteria: having more than 10 years' clinical experience and having given lectures or published articles regarding occupational therapy for people with dementia. We deliberately selected participants with various backgrounds to obtain a wide range of items for observing occupational engagement in theoretical sampling [20].

Prior to the interview, participants received written and oral explanations of the research purpose, research method, and protection of personal information. The explanation was carefully performed to ensure participant understanding, and written consent was obtained from each participant. The study was approved by the Ethics Committee of the Medical Department of Kyoto University (approval number: 1126).

### 2.2. Data Collection

Data were collected in a quiet room where the interview could be recorded face-to-face; only the participant and the interviewer were present. All interviews were audio-recorded with a voice recorder.

Interviews were conducted using an in-depth interview as a reference, according to the method outlined by Lyscak et al. [21]. The interview comprised open-ended questions in which information was solicited relating to the question, “What do you look for in clients with dementia when you assess effects of activity for them?” The primary goal of in-depth interviewing is to delve deeply into a particular event, issue, or context [21].

An unstructured, conversational data collection approach was used. During the interview, participants were asked to recall specific cases in which clients with dementia had experienced positive effects after having engaging in activities. In addition, it was explained to participants that activities were provided by occupational therapists to facilitate their clients to perform or participate in activities. Participants were encouraged to talk about and observe activities they performed with clients and how clients responded. Participants were also asked to explain how they determined that a specific activity was meaningful or effective for the client.

In cases where a participant could not recall a phenomenon, the interviewer asked additional questions to clarify what was observed. For example, the interviewer prepared specific prompts that were asked if necessary, such as “Please recall a situation where you tried to continue to provide a client with an activity. How did the client respond and why did you decide to provide that activity?”

Furthermore, to identify clients' concrete (i.e., objective) actions without the observer's subjective interpretation, follow-up questions were used to delve more deeply into participants' unconscious viewpoints regarding the observation. For instance, after a participant stated that a client's response had been different than his/her usual response when performing a specific activity, the interviewer asked, “How did he/she concretely respond in a manner different from the usual response?” The interviewer worded this question even more specifically if necessary.

Participants recalled and described one client until they could not recall anything further about him or her. Most participants recalled five clients. Each client's severity of dementia was assessed by having participants classify them into three categories: mild, moderate, or severe.

### 2.3. Data Analysis

Data analysis was performed according to Moustakas' modification of the Stevick-Colaizzi-Keen method for the analysis of phenomenological data [22]. All recorded interviews were transcribed verbatim. First, verbatim transcripts were read to gain a better understanding of the content, including which effects of activity were observed. In the next step of data analysis, participants' experiences of expressing effects of the activity were highlighted and key sentences were underlined and coded to extract invariant meaning units. From these units, similar codes were combined to generate higher-level abstracted themes. Data analysis was conducted using MAXQDA 10 (VERBI, GmbH, Berlin, Germany) qualitative data analysis software.

During data analysis, three researchers—who were experienced in qualitative analysis and were occupational therapists themselves—were asked to provide supervision on a regular basis and provide a second opinion to ensure the reliability and validity of the study. Themes, invariant meaning units, and the content of each theme were repeatedly checked until a consensus was reached.

## 3. Results

Participants consisted of 10 occupational therapists (6 males, 4 females) with an average clinical experience of 17.0 (±6.9 SD) years. Their primary clinical workplaces consisted of three nursing homes, three home-visiting rehabilitation centers, two hospitals specializing in dementia, one subacute rehabilitation hospital, one hospital for the elderly, and one preventive care service. Participant characteristics are shown in Table 1. Interview duration lasted between 42 and 67 min. From these interviews, 47 client stories were obtained and 154 labels were created. Of the 47 clients, 12 were classified as having mild dementia, 18 as moderate, and 17 as severe.

The viewpoints of occupational therapists while observing activities of patients with dementia were classified into five major themes and 18 subthemes. The major themes were “engaging activity,” “emotional expression during activity,” “verbal expression during activity,” “Social interaction through activity,” and “something obtained as outcome of activity.” Major themes, subthemes, and brief explanations are shown in Table 2. In the following section, subthemes are in italic font for clarity.

### 3.1. Major Theme 1: Engaging Activity

Participants observed clients'* directing attention to activity* and* initiating activity* as an effect of activities. Moreover,* continuing activity* and* concentrating on activity* led clients to devote themselves to activities. During activities, clients* expressed their skills and knowledge* or* tried new things during activity*, such as playing a role in an activity or trying new things until a solution was found.

#### 3.1.1. Directing Attention to Activity

Gazing at targets (e.g., book, TV, photo, and iPad) and responding to stimuli from an activity were observed as meaningful actions by some participants. For example, one client who had been previously inactive was watching images on television when her hometown was suddenly shown. When a location that was profoundly memorable to her appeared on the screen, she responded by opening her mouth and saying, “Ah!” The occupational therapist observed the client directing attention to the pictures and thus decided to show them to her once per week as an activity. Gazing at and responding to an activity stimulus indicated that the client was directing attention to an activity, leading therapists to judge that the activity was meaningful.

#### 3.1.2. Initiating Activity

Some participants noted whether a patient initiating an activity was important in assessing the patient. For example, a participant placed a magazine near an inactive client with severe dementia, and said to her, “This picture is so cute! This food looks yummy!” In response, the client willingly turned the page. The therapist was thus able to recognize the client's interest and ability for the first time. In another case, a client who usually roam around joined the group after a therapist called him saying, “It's time for art.” The participant observed an effective change in the client from a previously inactive condition to the initiation of activity (drawing a picture).

#### 3.1.3. Continuing Activity

Participants remarked that participation in activities often led patients to repeat the same activity and/or continue an activity. For instance, one participant found that an incommunicable client was interested in gardening. Therefore, the participant facilitated him in watering flowers. The participant could not determine whether the client was satisfied but observed that he kept watering for a long time, which seemed to be an effect of the activity. In this manner, repeating an activity or repeatedly spending a long time on an activity was often facilitated by engagement in the activity.

#### 3.1.4. Concentrating on Activity

Concentrating on an activity consisted of patients displaying serious facial expressions and settling down to do an activity without losing concentration. Participants observed clients' serious facial expressions during an activity, such as playing the samisen (a traditional Japanese musical instrument), cooking, and sewing. Furthermore, clients who were usually restless could participate in activities such as crafts and cooking. Based on their experiences, participants considered these activities to be effective.

#### 3.1.5. Expressing Skills and Knowledge

Expressing knowledge meant to naturally facilitate skills or knowledge that clients had acquired in the past. For example, one client weaved a basket out of willow shoots. The therapist considered her manipulation of the materials to be very skillful and therefore inferred that the client had prior experience weaving. Another example was when clients discussed knowledge of farm products while farming. Participants observed that expressing knowledge and skills often resulted from engaging in related activities.

#### 3.1.6. Trying New Things during Activity

Participants considered it meaningful when clients tried new things during activities. In one instance, a client was gardening while sitting in his wheelchair. He was unable to reach a seed and consequently stood up to reach it, demonstrating that he was trying something new. In another example, a client started drawing a picture using only one color but then noticed a case of colored pencils and started to use several colors. When clients solved problems and/or changed methods as per their own desires or necessity, therapists regarded this as a meaningful event.

### 3.2. Major Theme 2: Emotional Expression during Activity

Participants considered an activity to be effective when clients with dementia were found to* express delight* through smiling or acting/looking glad and* being calm*, for example, by appearing* relaxed* and anxiety-free.

#### 3.2.1. Expressing Delight during Activity

Many participants had observed clients smiling and being glad. Smiling was one of the main indicators therapists used to judge the effectiveness of activities. Expressing delight was divided into expression due to activities and expression caused by social interactions. For instance, some activities, such as knitting, singing, and watering flowers, made clients smile. As an example of expression caused from social interaction during activities, a client expressed gladness after receiving praise for her outstanding work on an artistic handicraft.

#### 3.2.2. Calm through Activity

Activities sometimes helped to soften clients' expression of emotion, especially for those who experienced anxiety, anger, restlessness, and desire to go home. The task of folding laundry was offered to a nursing home resident who frequently complained about wanting to go home and experienced restlessness, and the task of folding laundry became her purposeful activity in the nursing home. A staff in the nursing home thanked her and the resident then softened her expression. Gardening, cooking, and other activities helped to calm clients and ease their facial expression.

### 3.3. Major Theme 3: Verbal Expression during Activity

Participants suggested that verbal expression-related activities were meaningful as a viewpoint. This major theme was composed of two subthemes:* increased utterance* and* reminiscing*.

#### 3.3.1. Increased Utterance

Some participants paid attention to quantitative aspects of utterances when clients participated in activities. For example, a client with severe dementia who was otherwise nonverbal uttered one word (the name of her hometown) when she was asked about it during a group conversation. As another example, clients fluently mentioned their past experiences or confident topics. Participants regarded activities as effective when they led to increased numbers of positive utterances.

#### 3.3.2. Reminiscing

When performing an activity, patients sometimes recalled old memories. Some participants explained that reminiscing changed clients' facial expressions (e.g., leading them to smile) led them to feel satisfaction and/or to talk about something on their own accord. Although reminiscence itself was considered an activity, other activities led to reminiscing, which participants considered a result of participating in these activities.

### 3.4. Major Theme 4: Social Interaction through Activity

Social interaction resulting from engaging activities was considered crucial as a viewpoint. Five subthemes arose from this major theme:* initiating social interaction*,* sharing an activity with other people*,* teaching other people*,* considering other people*, and* expressing themselves to other people*.

#### 3.4.1. Initiating Social Interaction

Participants reported that engaging in an activity often led to the initiation of social interaction. For instance, while playing a game in a group, one client called out to another and encouraged her. Similarly, a client who was not interested in others gazed at another individual and started social interaction with them; this interaction had been inspired by a conversation that interested her. Initiating social interaction was thus regarded as an effect of participation in activities.

#### 3.4.2. Sharing an Activity with Other People

Sharing an activity with others was often observed during performing an activity. While participating in a game as part of a group, a client saw another member succeed and these two clients jointly experienced the joy of this individual's success. In another case, while painting a large picture, two clients shared roles with each other. Sharing something, such as a role, goal, feeling, instrument, or place, induced by performing activities with other people, was observed by some participants as an effect of participation in activities.

#### 3.4.3. Teaching Other People

One participant noted that an important aspect sometimes resulting from activities was that clients would pass on their knowledge or teach skills to others. For example, a nursing home client who had previously enjoyed sewing no longer engaged in this activity. The participant showed the client tools and materials for sewing and asked her, “Please, teach me to sew.” The client then began to sew and in doing so, the participant observed her talking, changing facial expressions, and laughing. Teaching an activity therefore provided patients with purpose and helped them to form social relationships. As such, this represented a positive effect of participation in an activity.

#### 3.4.4. Considering Other People

When clients performed activities, participants saw them consider the feelings of others. Clients were observed to worry about others, thank them, tell them a joke to calm them, or invite them to participate in activities. Some clients also gave others something they had made during an activity; for example, after cooking a meal, one client invited her friend to eat it. In sum, considering other people during or as a result of an activity was perceived as meaningful behavior by the participants.

#### 3.4.5. Expressing Themselves to Other People

Expressing clients' own opinions to others was regarded as being as important as considering other people. One participant mentioned that a client had insisted to others, “I want to paint red here. This part should be blue” and so forth. The participant believed that the activity held a special meaning for the client in this situation because he shared with others his own objectives and opinions regarding the activity.

### 3.5. Major Theme 5: Something Obtained as Outcome of Activity

The majority of participants observed that doing and finishing activities led to various outcomes for clients, enhancing their capability, satisfaction, and motivation to complete the following activity. This major theme was divided into three subthemes:* expressing a sense of capability*,* expressing satisfaction*, and enhancing appeal of* future activities*.

#### 3.5.1. Expressing a Sense of Capability

The verbal and facial expressions of participants were seen to indicate their capability after assessing the outcome, for example, to confirm the existence of themselves and to feel confident about their ability during and after an activity. For example, after completing a craft a patient took pride, saying to others, “This is my work.” As another example of confirmation of their own ability, after cooking one client verbally acknowledged, “There are things I can still do.” These behaviors were considered to represent a sense of capability as a result of participating in activities.

#### 3.5.2. Expressing Satisfaction

One effect that was reported by participants was that clients expressed satisfaction through their behavior as an outcome of activities and/or in response to praise from other people. In various activities, clients seemed to appreciate participating in a housekeeping activity such as folding laundry, were pleased by praise for their handmade crafts, and were delighted at winning a game.

#### 3.5.3. Enhancing Appeal of Future Activities

Participants considered it meaningful when clients engaged in planning future activities or started a new activity after the end of another. A successful activity empowered clients to start the next activity. In one example, a client looked forward to joining the same group to participate in a cooking activity the next week.

## 4. Discussion

The finding of this study summarized viewpoints expressed by occupational therapists to evaluate the effects of activities in clients with dementia. Referring to these subthemes could provide us with clear analytical viewpoints regarding engaging activities and help us to verbally interpret clients' meaningful behaviors. Categorizing these viewpoints was unconsciously used by well-experienced occupational therapists, which could be an important aspect in this study. Moreover, this framework which consisted of the subthemes has the potential to be used to assess clients' needs to engage in an activity and to examine the effects of an activity in people who cannot verbally express their needs or feelings, such as patients with dementia.

A novel aspect of this study was that well-experienced occupational therapists observed various aspects of interaction among people, activities, and the social environment. Certain observational assessments exist for individuals who cannot complete self-report questionnaires, such as the Volitional Questionnaire (VQ) [23]. The VQ provides the occupational therapist with insight into a person's inner motives related to participation in meaningful occupations. The VQ assesses individuals' volition, whereas the viewpoints gathered in this study involved individual strategies for managing an activity as well as feelings, emotions, and social interactions evoked by the activity. Engagement in activities requires that connectedness among self, others, and the environment is considered important to the individual [24]. Therefore, the viewpoints analyzed in this study could be used to widely assess and classify the effects of activity and to indicate to what degree a client can engage in an activity. These findings could be used to formulate new observational assessments of occupational engagement.

Two subthemes found in this study, reminiscing and calming though activity, could be specific to clients with dementia since reminiscence therapy is often used in this population. Calming is often required for individuals who display behavioral and psychological symptoms of dementia. Considering these subthemes, these viewpoints might be particular to clients with dementia. In other words, the other subthemes could be used to observe other clients without dementia. In particular, it might be useful to assess clients with cognitive disorder, such as children with severe dysfunction and people with stroke.

Hasselkus [25] performed a quantitative study to understand the experience of daycare staff for clients with dementia. This study found that daycare staff observed various indicators of clients' well-being, including smiling, laughing, being friendly, showing affection, not getting up consistently, not repeatedly asking questions, socializing with others, singing with “gusto,” and talking a lot. These indicators seemed to be effects of the activities performed by clients. For example, “smiling” in the previous study seems to be similar to “expressing delight during activity” as a subtheme in this study and “socializing with others” could be “social interaction through activity” as a major theme of this study. In other words, the subthemes found herein would be similar to and perhaps even more structured than these indicators of the previous study. Therefore, the viewpoints developed in the present study could be useful for understanding the perspective of individuals with dementia in regard to engagement in activities.

The occupational participation of nursing home residents with dementia has been found to be sporadic and passive [26]. A marked poverty of occupational engagement also exists in hospital and other care settings among clients with severe dementia. Occupations, which clients could self-choose, would provide them with feelings of autonomy, support identity, expression, and create personal space [27]. Therefore, if care staff and other rehabilitation staff share the viewpoints expressed by occupational therapists in the present study, healthcare staff in various domains could offer meaningful activities. This would in turn facilitate clients with dementia to increase occupational engagement and lead to a greater quality of life.

## Figures and Tables

**Table 1:** Participant characteristics.

Participant number	Clinical workplace	Gender	Years of experience
1	Nursing home	Female	19
2	Nursing home	Female	27
3	Care prevention	Male	13
4	Home-visiting rehabilitation	Female	14
5	Subacute rehabilitation hospital	Male	11
6	The hospital specializing in dementia	Female	20
7	Home-visiting rehabilitation	Female	30
8	Hospital for the elderly	Female	10
9	Home-visiting rehabilitation	Male	15
10	The hospital specializing in dementia	Male	11

**Table 2:** Major themes, subthemes, and brief explanations for each subthemes.

Main themes	Subthemes	Brief explanations for each subthemes
*Engaging activity*	Directing attention to activity	To direct one's attention and to show an interest to activity from a gaze, utterance, and an expression
	Initiating activity	To initiate an activity of one's own will or to initiate it after invitation without refusal to an activity
	Continuing activity	To continue an activity for a long time
	Concentrating on activity	To concentrate on an activity with serious expression and innocence
	Expressing skill and knowledge	To express knowledge or to explain it to others
	Trying new things during activity	To change the posture, or make a task and a role in an activity and to try a new thing during an activity

*Emotion during activity*	Expressing delight during activity	To smile or be glad without severe expression
	Being calm through activity	To be clam or relaxed without anxiety or poise

*Expression during activity*	Increasing utterance	To increase amount of words or to speak fluently
	Reminiscing	To recall the past and to do a reminiscence

*Social interaction through activity*	Starting social interaction	To be interested in others, for example, to gaze others and to try to communicate with them
	Sharing an activity with other people	To do something with others or to share place or activity with others
	Teaching other people	To engage in an activity as a role of teaching someone knowledge and skills
	Considering other people	To consider others, to do something for them, and to thank others during activity
	Expressing themselves to other people	To express one's feeling or opinion in words to others

*Something obtained as outcome of activity*	Expressing a sense of capability	To confirms one's own ability or to show sense of capability about results of the activity
	Expressing satisfaction	To indicate satisfaction to a result of the activity and praise from others
	Attracting next activities	To show the sign which looks forward to the next activity, to plan the next activity, or to begin different activity of one' will continuously

## Additional Points

*Study Limitations*. This study was the first attempt to gather viewpoints of well-experienced occupational therapists when observing clients engaged in activity. In this study, we interviewed occupational therapists regarding observational viewpoints for any activities in which their clients had participated, without identifying specific activities a priori. Occupational therapists utilize various activities in clinical settings. Therefore, certain subthemes were applicable to one activity but not another (e.g., an activity performed alone may not include social interaction). For these reasons, future studies will need to examine occupational therapists' observations according to purpose, form, or content of activity. Moreover, future studies could examine which of these viewpoints were suitable for each type of activity. Likewise, the viewpoints related to engaging activities are dependent on the severity of dementia. A client with severe dementia may not have many thoughts or ideas about carrying out an activity; he/she would be mainly concerned with sensation and with moving his/her body in response to those sensations [28]. The purpose of performing an activity is affected by the cognitive ability of the client. Therefore, future research is needed to clarify the relationship between the viewpoints and the severity of dementia. Finally, the study design purposefully included a variety of clinical settings and severities of dementia in order to encompass various viewpoints. Although 47 cases are not considered small for qualitative research, the sample consisted of only ten participants. Thus, all occupational therapists might not agree with the findings of this study. Future studies must examine the opinions of other occupational therapists regarding the viewpoints collected herein, which will in turn lead to the validation of these viewpoints.
